# The Paravascular Pathway for Brain Waste Clearance: Current Understanding, Significance and Controversy

**DOI:** 10.3389/fnana.2017.00101

**Published:** 2017-11-07

**Authors:** Andrew Bacyinski, Maosheng Xu, Wei Wang, Jiani Hu

**Affiliations:** ^1^School of Medicine, Wayne State University, Detroit, MI, United States; ^2^Department of Radiology, The First Affiliated Hospital of Zhejiang Chinese Medical University, Hangzhou, China; ^3^Department of Radiology, Wayne State University, Detroit, MI, United States

**Keywords:** paravascular pathway, glymphatic system, perivascular pathway, brain waste clearance, amyloid-beta

## Abstract

The paravascular pathway, also known as the “glymphatic” pathway, is a recently described system for waste clearance in the brain. According to this model, cerebrospinal fluid (CSF) enters the paravascular spaces surrounding penetrating arteries of the brain, mixes with interstitial fluid (ISF) and solutes in the parenchyma, and exits along paravascular spaces of draining veins. Studies have shown that metabolic waste products and solutes, including proteins involved in the pathogenesis of neurodegenerative diseases such as amyloid-beta, may be cleared by this pathway. Consequently, a growing body of research has begun to explore the association between glymphatic dysfunction and various disease states. However, significant controversy exists in the literature regarding both the direction of waste clearance as well as the anatomical space in which the waste-fluid mixture is contained. Some studies have found no evidence of interstitial solute clearance along the paravascular space of veins. Rather, they demonstrate a perivascular pathway in which waste is cleared from the brain along an anatomically distinct perivascular space in a direction opposite to that of paravascular flow. Although possible explanations have been offered, none have been able to fully reconcile the discrepancies in the literature, and many questions remain. Given the therapeutic potential that a comprehensive understanding of brain waste clearance pathways might offer, further research and clarification is highly warranted.

## Introduction

In peripheral tissues, the lymphatic system functions to clear proteins solutes and metabolic wastes from the interstitial spaces between cells (Ellis, 2006). The brain parenchyma lacks a typical, histologically identifiable lymphatic system, which is curious given the brain’s high metabolic rate and sensitivity to alterations in its extracellular space (Abbott, 2004). For decades, it was generally understood that waste substances in the central nervous system (CNS) were cleared to cerebrospinal fluid (CSF) by convective bulk flow of interstitial fluid (ISF) coursing diffusely through brain parenchyma (Cserr et al., 1981; Abbott, 2004; Sykova and Nicholson, 2008), draining into peripheral lymphatics of the neck to ultimately reach systemic circulation (Bradbury et al., 1981; Bradbury and Westrop, 1983). However, some early studies argued that, rather than a diffuse process, solute transport occurred via an anatomically and functionally discrete space surrounding the blood vessels of the brain (Földi et al., 1968; Rennels et al., 1985, 1990). This space, termed the paravascular space, was later identified as a possible route for the clearance of glucose, lactate and amyloid beta (Ball et al., 2010).

Recently, a team of researchers headed by Iliff and Nedergaard fully characterized the paravascular space as a pathway for the clearance of interstitial solutes. By tracking the movement of small fluorescent tracers, they showed that CSF enters the brain parenchyma along para-arterial spaces, mixes with ISF and solutes, and follows para-venous spaces to ultimately be removed from the brain. This paravascular pathway was termed the “glymphatic” system due to its dependence on glial cells and functional similarity to the peripheral lymphatic system (Iliff et al., 2012).

Since its discovery, significant research has been conducted on the paravascular pathway, further elucidating its physiological function and possible role in disease. However, disagreement still exists in the literature regarding the structures involved in interstitial waste clearance and the direction of fluid flow. This brief review article will examine the anatomy, function and clinical significance of the paravascular pathway, as well as the controversy associated with it. An examination of what is currently known and what remains to be clarified may provide inspiration for future research on this clinically important topic.

## Anatomy of the Spaces Surrounding the Cerebral Vasculature

**Figure 1:**
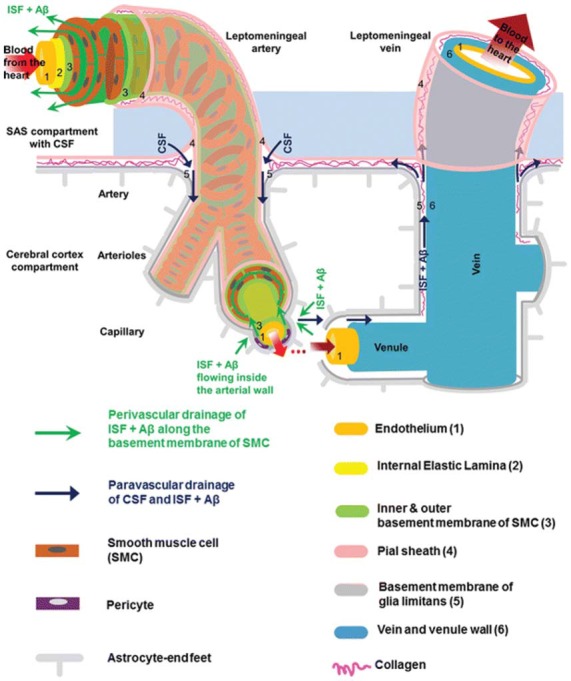
Anatomy of the paravascular and perivascular spaces. The arterial paravascular space is bounded internally by the pial sheath **(4)** and externally by the basement membrane of glia limitans **(5)**. The venous paravascular space is bounded internally by the vein wall **(6)** and externally by the glia limitans. The perivascular space is found within the middle layers of the basement membrane of arterial smooth muscle cells **(3)**. No perivascular space is present in venous vasculature. In each space, the direction of fluid flow and waste clearance, such as amyloid beta (Aβ), is depicted with arrows. Reprinted with permission © Bakker et al. (2016).

The brain is vascularized by the anterior and posterior cerebral circulation (Prince and Ahn, 2013). At the surface of the cortex, cerebral arteries form pial arteries, which extend through the subarachnoid and subpial spaces. Leptomeningeal cells forming a pial sheath are reflected from the surface of the brain to coat these arteries and veins in the CSF-containing subarachnoid space (Weller, 2005; Bakker et al., 2016). As pial arteries enter the brain parenchyma, they transition into penetrating arterioles with a surrounding paravascular space, known as the Virchow-Robin space (Zhang et al., 1990). This donut-shaped space surrounding the vasculature contains CSF and is bounded by a pial sheath internally and the basement membrane of astrocytic endfeet, known as the glia limitans, externally (Figure 1; Weller et al., 2009; Jessen et al., 2015; Bakker et al., 2016). Recent evidence suggests that, in rodent models, no pial sheath is found at the internal boundary of the paravascular space (Bedussi et al., 2017), and this space forms a continuous fluid compartment with the subarachnoid space (Bedussi et al., 2015). However, as reviewed by Brinker et al. (2014) there may be structural differences between rodents and humans regarding the continuity of the paravascular and subarachnoid spaces.

Another space exists within the arterial tunica media, between the middle layers of the basement membrane of arterial smooth muscle cells (Figure 1). This structure, known as the perivascular space, is also a conduit for fluid flow. Multiple studies have demonstrated its involvement in ISF and solute flux from the brain parenchyma to the cervical lymphatic system in a direction opposite to that of blood flow (Carare et al., 2008; Weller et al., 2009; Abbott, 2013; Hawkes et al., 2014; Bakker et al., 2016; Morris et al., 2016; Ueno et al., 2016). Retrograde fluid flow through this space forms the crux of the controversy surrounding the paravascular pathway and will be discussed later.

As penetrating arterioles dive deeper into the parenchyma and narrow to form capillaries, the smooth muscle layer and pial sheath are abolished, and both the paravascular and perivascular spaces become continuous with the endothelial basal lamina (Morris et al., 2016). This structure is a thin matrix derived from endothelial and astrocytic elements (Bakker et al., 2016; Morris et al., 2016). As capillaries continue to form venules and veins, this fluid-filled space expands and becomes bounded by the venous wall internally and the glia limitans externally (Figure 1). Thus, the paravascular space is a continuous compartment from arteries to capillaries to veins.

## Proposed Model for Glymphatic Fluid Flow

**Figure 2:**
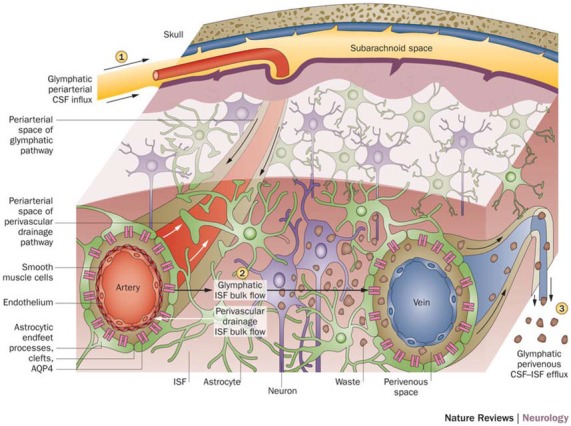
Schematic of glymphatic and perivascular waste clearance routes. According to the glymphatic model, cerebrospinal fluid (CSF) flows along para-arterial spaces **(1)**, mixes with interstitial fluid (ISF) and solutes **(2)**, and is cleared from the brain along para-venous spaces **(3)**. Efficient glymphatic clearance of waste and solutes is dependent on fluid movement across aquaporin-4 (AQP4) channels located on astrocytic endfeet surrounding the parenchymal vasculature. In contrast, perivascular drainage of ISF and solutes occurs along the middle layers of the basement membrane of arterial smooth muscle cells and, as indicated by arrows, flows in a direction opposite to that of glymphatic clearance. Reprinted with permission from Macmillan Publishers Ltd: © Tarasoff-Conway et al. (2015).

With a working anatomical understanding of the compartments surrounding the cerebral vasculature, we can now turn our attention to the model of paravascular fluid flow. As described, the glymphatic system is composed of a unidirectional current of fluid flowing through the paravascular space of penetrating arteries and arterioles to that of large caliber parenchymal draining veins (Iliff et al., 2012). The schematic, seen in Figure 2, shows CSF entering the brain along the Virchow-Robins space and flowing centrally into brain parenchyma within the para-arterial compartment and capillary basal lamina. Due to the loose composition of the basal lamina, there is little resistance to CSF influx (Jessen et al., 2015). Large solutes (>100 kDa) present in CSF that lack a specific molecular transport pathway are unable to pass through the 20–50 nm clefts that separate astrocytic endfeet of the glia limitans and are constrained to this space, flowing along the capillary basal lamina as it becomes continuous with the perivenous space (Iliff et al., 2013; Jessen et al., 2015). CSF water, ions, and small solutes (<100 kDa) may enter the interstitial space through aquaporin-4 (AQP4) channels, ion transporters or channels, and astrocytic endfeet clefts, respectively (Iliff et al., 2012, 2013; Hubbard et al., 2015; Jessen et al., 2015). After mixing with ISF and interstitial solutes, this fluid re-enters the paravascular space of draining veins by similar mechanisms. From this space, waste may ultimately be cleared from the brain by draining into cervical lymphatics, dispersing into the subarachnoid CSF, or crossing the vasculature to enter the bloodstream (Iliff et al., 2012; Tarasoff-Conway et al., 2015; Louveau et al., 2016). Additionally, lymphatic vessels lining the dural sinuses have recently been identified (Louveau et al., 2015). These meningeal vessels may play an important role in draining glymphatic fluid and solutes to deep cervical lymph nodes and other peripheral structures.

Consistent with previous observations (Cserr et al., 1981; Geer and Grossman, 1997; Abbott, 2004), it was postulated by Iliff and Nedergaard that the transport of CSF and solutes through the glymphatic pathway was driven by convective bulk flow, which is characterized by the concomitant movement of water and solutes due to changes in pressure gradients (Iliff et al., 2012). They hypothesized that high pressure arterial pulsations, combined with low pressure paravenous spaces and increased expression of AQP4 in paravenous astrocytic endfeet, may create an arteriovenous hydrostatic gradient that drives paravascular CSF bulk flow and ISF clearance (Iliff et al., 2012). Further evidence suggests that respiration, vasomotor wave fluctuations, and body posture may also contribute to this mechanism (Lee et al., 2015; Kiviniemi et al., 2016). Recently, however, the bulk flow hypothesis has been challenged (Hladky and Barrand, 2014; Asgari et al., 2016). Future studies investigating the forces driving fluid flow through the paravascular pathway are expected.

## Function and Significance of the Paravascular Pathway

The glymphatic system allows for intimate exchange between CSF and ISF, and is involved in the delivery of nutrients to brain parenchyma and interstitial waste clearance (Matsumae et al., 2016). Recent studies have demonstrated that apolipoprotein E, small lipid molecules and signaling molecules may be delivered from CSF to the brain parenchyma via the glymphatic pathway (Rangroo Thrane et al., 2013; Achariyar et al., 2016). ApoE, a compound highly expressed by the choroid plexus and the predominant apolipoprotein in CSF, is a major carrier of cholesterol and is essential for neuron function and plasticity. Although this lipoprotein is locally produced by astrocytes, glymphatic transport of CSF-derived ApoE may facilitate a wider distribution in the brain parenchyma. It is likely that the glymphatic pathway also contributes to the distribution of other growth factors, neuromodulators, carrier proteins, and nutrients (Kress et al., 2014). Additionally, the paravascular system might serve as a useful pathway for drug delivery to the brain (Hadaczek et al., 2006).

It is well established that the glymphatic system participates in the removal of amyloid beta (Aβ), a pathological hallmark of Alzheimer disease, from the brain (Tarasoff-Conway et al., 2015; Simon and Iliff, 2016). Iliff and Nedergaard originally demonstrated that the clearance of radiolabeled Aβ injected into brain parenchyma was cleared via paravascular routes. Moreover, they found that this process was dependent on AQP4, as AQP4-knockout mice showed a 55% reduction in Aβ clearance compared to controls (Iliff et al., 2012). A later study explored Aβ clearance in the aging brain and found a 40% reduction in old mice compared to young, accompanied by decreased arterial pulsatility and AQP4 expression (Kress et al., 2014). Other studies have found decreased glymphatic Aβ clearance in various disease state models, including sleep deprivation (Xie et al., 2013), depression (Xia et al., 2017) and obstructive sleep apnea (Ju et al., 2016). In contrast, exercise (He et al., 2017) and omega-3 fatty acids (Ren et al., 2017) have been associated with improved glymphatic Aβ removal. Not surprisingly, dysfunction of the paravascular pathway may serve as an early biomarker of Alzheimer disease (Peng et al., 2016), and novel methods of evaluating glymphatic function are currently being developed (Yang et al., 2013; Eide and Ringstad, 2015; Huffman et al., 2016; Kiviniemi et al., 2016; Ramirez et al., 2016; Rivera-Rivera et al., 2016; Taoka et al., 2017).

In addition to Aβ clearance, the glymphatic system may be involved in the removal of other interstitial solutes and metabolites. By measuring the lactate concentration in the brains and cervical lymph nodes of awake and sleeping mice, Lundgaard et al. (2017) demonstrated that lactate can exit the CNS via the paravascular pathway. Their analysis took advantage of the substantiated hypothesis that glymphatic function is promoted during sleep (Xie et al., 2013; Lee et al., 2015; Liu et al., 2017). In another study, Iliff et al. (2014) showed that tau, a protein implicated in progressive neurodegenerative diseases, is also cleared by this pathway. Moreover, they demonstrated that glymphatic function is impaired after traumatic brain injury, which is clinically relevant given that common biomarkers for this insult are transported to the peripheral blood via the paravascular pathway (Plog et al., 2015). There is also evidence that alpha-synuclein, a protein involved in neurodegenerative diseases such as Parkinson’s disease, may also be cleared, and possibly spread, by this pathway (Valdinocci et al., 2017).

As dysfunction of the glymphatic system has been implicated in many disease states, including neurodegenerative disease (Iliff et al., 2012, 2014), microinfarcts (Venkat et al., 2017; Wang et al., 2017), migraines (Schain et al., 2017), diabetes (Jiang et al., 2017) and glaucoma (Wostyn et al., 2017), further research on this pathway is highly anticipated. However, the literature suggests that the glymphatic system may not be the only route for waste clearance in the brain. Multiple studies have shown that a perivascular pathway plays an important role in the drainage of ISF and solutes to peripheral structures.

## Controversy: the Perivascular Pathway

Evidence for a periarterial pathway for brain waste clearance was demonstrated as early as 1984. Szentistványi et al. (1984) discovered that when tracer was injected into the brain parenchyma of mice, it subsequently was found in the periarterial space of major cerebral arteries supplying the tissue. Interestingly, the measured concentration of tracer was much higher within the periarterial space than the surrounding CSF, and no tracer was found along draining veins. In 2008, similar results were demonstrated by Carare et al. (2008), who used confocal microscopy to visualize tracer in the basement membrane of capillaries and within the tunica media of penetrating arteries following injection into the gray matter of the caudate and putamen in mice. These results have been verified and expounded upon in more recent experiments (Arbel-Ornath et al., 2013; Morris et al., 2016), further lending evidence to the perivascular hypothesis.

The current model for the perivascular pathway depicts ISF and solutes entering the basement membrane of capillaries and following the tunica media of penetrating arteries within the basal lamina of smooth muscle cells. Curiously, solute clearance via the perivascular pathway is in a direction opposite to that of paravascular flow and blood flow (Figure 2). From the peri-arterial space, solute may ultimately be cleared from the brain by dispersing in CSF (Zhang et al., 1992; Kida et al., 1993; Carare et al., 2008) or directly emptying into peripheral lymphatic tissue surrounding the large arteries of the neck (Szentistványi et al., 1984; Shinkai et al., 1995; Weller, 2005).

The direction of fluid flow described by the perivascular and paravascular models seems to be the foundation of the controversy in the literature, and may be partially explained by methodological differences between studies. Bakker et al. (2016) note that, historically, most studies in which tracer was injected into CSF resulted in paravascular flow, while studies in which tracer was injected into brain parenchyma resulted in perivascular flow. Pressure and volume disturbances from injected tracer (Hladky and Barrand, 2014), compounded with dispersion forces caused by arterial pulsations (Asgari et al., 2016), may account for these differences. In their 2012 study, Iliff and Nedergaard maintained that the small amount of tracer Aβ observed in the periarterial space surrounding the parenchymal injection site was an artifact and did not reflect the natural pathway for solute efflux. In contrast to other studies, they showed that most of the labeled Aβ was localized around capillaries and draining veins following parenchymal injection, demonstrating paravascular flow (Iliff et al., 2012). However, other studies have demonstrated that both labeled and native Aβ is predominantly distributed in the walls of arteries, not veins (Hawkes et al., 2011, 2014). Is there a different explanation?

Another possibility that may account for the discrepancies noted in various studies is that the pathways are in fact two distinct systems functioning simultaneously (Morris et al., 2016). At the level of the penetrating arterioles the paravascular and perivascular spaces are separate, and fluid flow through one space does not necessarily preclude flow through the other. A comprehensive and recent review by Hladky and Barrand (2014) argues that the two pathways are not mutually exclusive, and both may contribute to interstitial solute clearance depending on the conditions and local environment within the brain. However, other authors have been critical of this idea, suggesting that the concomitant existence of the two pathways would make little sense physiologically, given the opposing directions of fluid flow, especially at the level of the capillary where only one compartment is present (Bakker et al., 2016). Furthermore, a recent study found no pial sheath separating the perivascular and paravascular spaces in mice (Bedussi et al., 2017), suggesting that the two spaces may be functionally continuous. If this is true in humans, it is unlikely that both pathways would function simultaneously under physiological conditions.

Obviously, many questions concerning the two pathways require further elucidation. Are they distinct compartments in humans or anatomically and functionally continuous? Does normal interstitial solute clearance follow a periarterial route, a paravenous route, or both? Does this process change with disease? What are the driving forces behind these pathways? Further research and clarification is highly anticipated.

## Summary

In conclusion, the paravascular pathway is a recently characterized system for waste clearance in the brain. Since its discovery, a growing body of evidence has demonstrated the importance of this discrete system in metabolite and protein removal and, to a lesser extent, nutrient delivery. However, discrepancies in the literature suggest that there may be other mechanisms and complexities by which waste products are cleared to peripheral tissues. A comprehensive understanding of the routes by which brain-derived pathologic proteins and wastes are transported may give insight into the pathophysiology and treatment of neurodegenerative disease. While no explanation to date has been able to fully account for the inconsistencies noted in the literature, the studies reviewed in this article lay a solid foundation for future inquiry and discussion.

## Author Contributions

All the authors contributed substantially to the work, including the concept, literature review, manuscript drafting and final review.

## Conflict of Interest Statement

The authors declare that the research was conducted in the absence of any commercial or financial relationships that could be construed as a potential conflict of interest. The reviewer MS and handling Editor declared their shared affiliation.
